# IGF-I: A Key Growth Factor that Regulates Neurogenesis and Synaptogenesis from Embryonic to Adult Stages of the Brain

**DOI:** 10.3389/fnins.2016.00052

**Published:** 2016-02-23

**Authors:** Vanesa Nieto-Estévez, Çağla Defterali, Carlos Vicario-Abejón

**Affiliations:** ^1^Consejo Superior de Investigaciones Científicas (CSIC), Instituto CajalMadrid, Spain; ^2^Centro de Investigación Biomédica en Red sobre Enfermedades Neurodegenerativas (CIBERNED)Madrid, Spain

**Keywords:** IGF-I, neurogenesis, proliferation, survival, differentiation, maturation, migration, IGF-IR

## Abstract

The generation of neurons in the adult mammalian brain requires the activation of quiescent neural stem cells (NSCs). This activation and the sequential steps of neuron formation from NSCs are regulated by a number of stimuli, which include growth factors. Insulin-like growth factor-I (IGF-I) exert pleiotropic effects, regulating multiple cellular processes depending on their concentration, cell type, and the developmental stage of the animal. Although IGF-I expression is relatively high in the embryonic brain its levels drop sharply in the adult brain except in neurogenic regions, i.e., the hippocampus (HP) and the subventricular zone-olfactory bulb (SVZ-OB). By contrast, the expression of IGF-IR remains relatively high in the brain irrespective of the age of the animal. Evidence indicates that IGF-I influences NSC proliferation and differentiation into neurons and glia as well as neuronal maturation including synapse formation. Furthermore, recent studies have shown that IGF-I not only promote adult neurogenesis by regulating NSC number and differentiation but also by influencing neuronal positioning and migration as described during SVZ-OB neurogenesis. In this article we will revise and discuss the actions reported for IGF-I signaling in a variety of *in vitro* and *in vivo* models, focusing on the maintenance and proliferation of NSCs/progenitors, neurogenesis, and neuron integration in synaptic circuits.

## Insulin-like growth factor I (IGF-I)

IGF-I belongs to the insulin family which is divided in two groups of peptides: one includes insulin and the IGFs and the other relaxin and insulin-like hormones. In the insulin group, each peptide binds to a specific receptor with high affinity, although it can also bind to the other receptor with low affinity (Table 1). Furthermore, the insulin receptor and the IGF-I receptor (IGF-IR) can form heterodimers with similar affinity for both growth factors (Hernández-Sánchez et al., 2008). The IGF-IR has the higher affinity for IGF-I but its affinity is 10 times lower for IGF-II and 250 times lower for insulin (Versteyhe et al., 2013). In addition, there are at least seven IGF-binding proteins (IGFBPs) that increase the half-life of the peptide by preventing its proteolysis and modulating the interaction with the receptor (Table 1; Ocrant et al., 1990; Hwa et al., 1999; Bondy and Cheng, 2004; Agis-Balboa et al., 2011; Fernandez and Torres-Alemán, 2012; Agis-Balboa and Fischer, 2014).

**Table 1:** **The insulin group of growth factors**.

**Ligand**	**Receptor**	**Binding proteins**
Insulin	Insulin receptor (IR: high affinity) and IGF-IR (low affinity)	Not known
IGF-I	IGF-IR (high affinity), IR (low affinity), and IGF-IIR (very low affinity)	IGFBP1–5 (high affinity) and IGFBP6-7 (low affinity)
IGF-II	IGF-IIR (high affinity), IGF-IR (low affinity), and IR (very low affinity)	IGFBP6-7 (high affinity) and IGFB1–5 (low affinity)

The mature IGF-I is a single polypeptide chain of 70 amino acids (7.5 kDa) with 57 amino acids being identical in mammals, birds, and amphibians (Liu et al., 1993; Russo et al., 2005; Annunziata et al., 2011). The IGF-IR is a tyrosine kinase receptor characterized by tetramers, which are composed of two α subunits and two β subunits (Russo et al., 2005; Annunziata et al., 2011; Vogel, 2013).

## Expression of IGF-I and IGF-I receptor

IGF-I is abundantly produced during embryonic development in many tissues, but its expression is markedly reduced during postnatal life. In the adult individual, IGF-I is mainly synthesized in the liver via a process regulated by the growth hormone (GH). Furthermore, there is a small local production in tissues including brain regions such as the SVZ, the OB, the HP, and the cerebellum (CB; Rotwein et al., 1988; Ye et al., 1997). In the brain, IGF-I can be synthesized by neurons independently of GH action (Bartlett et al., 1991, 1992; Bondy and Cheng, 2004; Russo et al., 2005; Fernandez and Torres-Alemán, 2012). Systemic IGF-I can pass from the blood to the cerebrospinal fluid through the lipoprotein receptor-related protein 2 (LRP2). In addition, IGF-I can cross the blood-brain-barrier by binding to the IGF-IR present on endothelial cells and later it is picked up either by astrocytes to be transferred to neurons or directly by neurons (Nishijima et al., 2010; Fernandez and Torres-Alemán, 2012). Therefore, IGF-I can act in the brain in an endocrine, paracrine or autocrine manner.

IGF-IR expression begins early during embryonic development in regions that include the cortex, OB, HP, CB, hypothalamus (HT), and spinal cord (Bondy et al., 1990). Postnatally, IGF-IR levels are slightly reduced and in the adult, its expression is clearly detected in the SVZ, OB, HP, CB, and the choroid plexus (Bondy and Cheng, 2004; Russo et al., 2005; Fernandez and Torres-Alemán, 2012).

## IGF-I/IGF-IR signaling pathways

The specific IGF-I binding to IGF-IR triggers the auto phosphorylation of the receptor and the activation of the insulin receptor substrates (IRS). These activated IRSs are auto-phosphorylated and in turn activate the intracellular signaling pathways including PI3K and MAP kinase pathways (Liu et al., 1993; Bondy and Cheng, 2004; Bateman and McNeill, 2006; Fernandez and Torres-Alemán, 2012; Puche and Castilla-Cortázar, 2012).

The phosphatidylinositol 3-kinase (PI3K) phophorylates the serine/threonine protein kinase (Akt) through the phosphoinositide-dependent protein kinase (PDK). Phospho-Akt promotes the translocation of the glucose transporters to the plasma membrane affecting cell metabolism (Bondy and Cheng, 2004; Fernandez and Torres-Alemán, 2012). Another Akt substrate is the mammalian target of rapamycin (mTOR). mTOR1 activates p70S6K, regulating protein synthesis while mTOR2 activates a series of kinases (including Akt), affecting proliferation, cell migration, and positioning (Hurtado-Chong et al., 2009; Iwanami et al., 2009; Onuma et al., 2011; Fernandez and Torres-Alemán, 2012; Paliouras et al., 2012; Pun et al., 2012). Akt also promotes the activation of fork head transcription factor (FOXO), which regulates cell proliferation, oxidative stress and apoptosis (Bateman and McNeill, 2006; Fernandez and Torres-Alemán, 2012; O'Kusky and Ye, 2012). Moreover, Akt can activate the cAMP responsive element binding protein (CREB) regulating the transcription of genes involved in cell cycle progression, survival, and differentiation. The binding of IGF-I to the IGF-IR can also promote the activation of Son of sevenless (SOS) triggering the phosphorylation of RAS, which in turn promotes the activation of MAPK. Later, MAPK produces the phosphorylation of ERK inducing proliferation of multiple cell types (Baltensperger et al., 1993; Skolnik et al., 1993; Bateman and McNeill, 2006; Cundiff et al., 2009; Fernandez and Torres-Alemán, 2012).

## IGF-I functions

### Body and organ growth

IGF-I is a pleiotropic factor involved in multiple processes, so its actions are different depending on its concentration, the cell type, and the developmental stage of the animal.

IGF-I is necessary very early during pregnancy because it promotes embryo implantation in the uterus (O'Kusky and Ye, 2012). Later, IGF-I is important for the proper prenatal growth and postnatal survival of the animal. This fact is reflected in the smaller size of the global *Igf-I* Knockout (KO) mice and *Igf-Ir* KO mice compared to their control littermates after birth. The liver-specific *Igf-I*-deficient (LID) mice have a similar body size compared to the control animals, suggesting that IGF-I affects tissue growth in an autocrine or paracrine manner (Yakar et al., 2002). Interestingly, exogenously administrated IGF-I can compensate for most autocrine/paracrine actions of this growth factor (Wu et al., 2009). The large majority of global *Igf-I* KO mice die soon after birth due to insufficient lung maturation, although the death rate depends on the mouse strains (Liu et al., 1993; Moreno-Barriuso et al., 2006; Kappeler et al., 2008; Hurtado-Chong et al., 2009; Pais et al., 2013). The muscles, brain, bones and skin are affected by the lack of IGF-I, as reflected by the muscle hypoplasia and the reduced brain size, ossification, and skin thickness found in the KO mice (Baker et al., 1993; Liu et al., 1993; Powell-Braxton et al., 1993; Beck et al., 1995; Pichel et al., 2003). This phenotype is also observed in the few surviving postnatal KO mice which show a reduction in body and brain size, lower development of ossification centers, infertility, and deafness (Baker et al., 1993; Wang et al., 1999; Yakar et al., 2002; Fernández-Moreno et al., 2004; Cediel et al., 2006; Stratikopoulos et al., 2008; Hurtado-Chong et al., 2009; Wu et al., 2009; O'Kusky and Ye, 2012; Rodríguez-de la Rosa et al., 2015).

In humans, mutations in the *IGF-I* and *IGF-IR* genes cause growth retardation including microcephaly (Roback et al., 1991; Woods et al., 1996; Walenkamp et al., 2005, 2013; van Duyvenvoorde et al., 2010; Burkhardt et al., 2015). Furthermore, the congenital deficiency of *IGF-I* or *IGF-IR* are features of the Laron syndrome, which also includes growth hormone receptor (GHR) deficiency and/or the alteration of molecules of the GH and IGF-I signaling pathways. Patients with this syndrome experience less growth after birth and this becomes more severe with age, leading to smaller brain size, smaller heart, less muscle development, among other deficits (Puche and Castilla-Cortázar, 2012). Although short stature is a common feature of the individuals bearing *IGF-I* and/or *IGF-IR* mutations, a recent study has described intragenic deletions of the *IGF-IR* associated to a developmental delay and intellectual disability of five people that do not have a significant short stature (Witsch et al., 2013).

**Figure 1:**
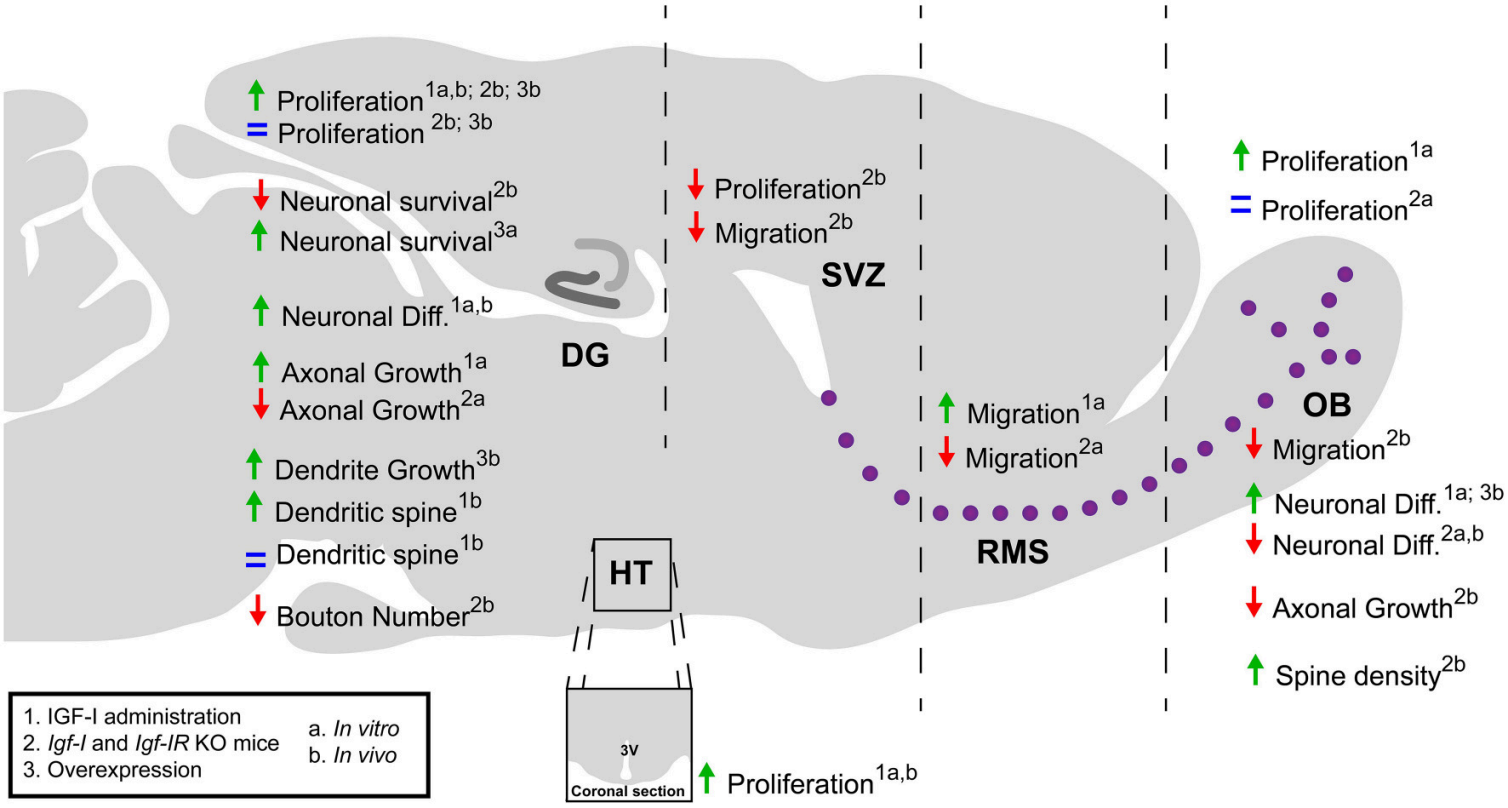
**A schematic summary of the role of IGF-I during postnatal-adult neurogenesis**. The IGF-I is a pleiotropic factor that affect a variety of cellular processes. The administration of IGF-I enhances cell proliferation and neurogenesis in the OB, DG, and HT *in vitro* and *in vivo*. However, the use of transgenic mice that overexpress IGF-I or lack the *Igf-I* and *Igf-IR* genes has revealed contradictory effects of IGF-I signaling on these processes. IGF-I promotes cell migration in the SVZ-OB and the survival and axonal growth of neurons in both the SVZ-OB and DG. In contrast, the effects on dendritic spines and synapse formation may depend on the neuron type and developmental stage of the cell and animal. DG, dentate gyrus; Diff., differentiation; OB, olfactory bulb; RMS, rostral migratory stream; SVZ, subventricular zone; HT, hypothalamus; 3V, third ventricle.

During central nervous system (CNS) development and adult neurogenesis, the IGF-I/IGF-IR system regulates the proliferation and survival of neural progenitors, as well as the generation, differentiation, and maturation of neurons in multiple ways (Beck et al., 1995; Cheng et al., 2001; Pichel et al., 2003; Russo et al., 2005; Hurtado-Chong et al., 2009; Fernandez and Torres-Alemán, 2012; O'Kusky and Ye, 2012; Chaker et al., 2015). These aspects are discussed in depth below (Figure 1).

## Cell proliferation

IGF-I promotes proliferation of neural cells by interacting with the IGF-IR which may activate the PI3K/Akt or the MAP kinase pathways (Otaegi et al., 2006; Mairet-Coello et al., 2009; Vogel, 2013; Yuan et al., 2015). During embryonic development, IGF-I promotes the proliferation of neuroepithelial progenitor cells both *in vitro* (Hernández-Sánchez et al., 1995; Arsenijevic et al., 2001; Vicario-Abejón et al., 2003; Cui and Almazan, 2007; Magariños et al., 2010; Ziegler et al., 2012) and *in vivo* (Popken et al., 2004; Ye and D'Ercole, 2006; Hu et al., 2012). This positive effect of IGF-I on cell proliferation was also observed postnatally and in the adult brain (Aberg et al., 2000, 2003; Trejo et al., 2001; Gago et al., 2003; Popken et al., 2004; Kalluri et al., 2007; Kouroupi et al., 2010; Pérez-Martín et al., 2010; Yuan et al., 2015), although enhanced proliferation was not found in the adult HP of an astrocyte-conditional IGF-I overexpressing mouse (Carlson et al., 2014). In contrast, mice that overexpress IGF-I under the regulation of the Nestin promoter, active in neural progenitors, show an increase in brain size both at E18 and postnatally (Popken et al., 2004) due to a shorter cell cycle produce by the decrease in the G1 phase length (Hodge et al., 2004).

During OB development, IGF-I can stimulate the proliferation of stem and progenitor cells as observed in embryonic olfactory bulb stem cells (eOBSCs) cultures where the addition of IGF-I increases the number of proliferative cells and of neurospheres compared to untreated cultures. However, when eOBSCs were isolated from *Igf-I* KO embryos, there was no difference in the percentage of BrdU^+^ cells compared to wildtype (WT) cells (Vicario-Abejón et al., 2003). In contrast, a decrease in the number of cells in the M phase of cell cycle was observed in the SVZ of *Igf-I* adult KO mice (Hurtado-Chong et al., 2009; Figure 1).

IGF-I also affects cell proliferation in the dentate gyrus (DG) of the HP (Figure 1). In fact, in cultures of adult rat DG progenitor cells an increase in the number of dividing cells was found after IGF-I treatment (Aberg et al., 2003). In addition, when mice were administered with IGF-I peripherally, more BrdU^+^ cells in the DG were detected (Aberg et al., 2000). A similar effect was observed after physical exercise, a condition that enhances IGF-I entry into the brain (Trejo et al., 2001, 2008; Glasper et al., 2010; Fernandez and Torres-Alemán, 2012). However, in the *Igf-I* KO mice the lack of IGF-I produced an increase in the number of proliferative cells in DG (Cheng et al., 2001) whereas both GH/IGF-I deficiency and deleting the IGF-IR in neural progenitors did not specifically affect proliferation in the postnatal-adult DG (Lichtenwalner et al., 2006; Liu et al., 2009).

In sum, the majority of these findings have shown that an increase in the IGF-I levels promotes cell proliferation both *in vitro* and *in vivo*. However, deleting this growth factor and/or its receptor in KO mouse has produced contrasting effects that are not completely elucidated yet (Figure 1). The expression of insulin, IGF-II and of truncated IGF-I-related peptides in the KO mice might partially explain the discrepancy obtained in different studies. Although the majority of truncated peptides are thought to be non-functional, we cannot completely exclude that in certain KO mice lines they could affect the results described. The development of new technologies such as the CRISPR-Cas9 system, which allows the complete deletion of specific genes, and the generation of double or triple KO mouse lines could help to understand the effect of the deletion of IGF-I or its receptor in cell proliferation during adult neurogenesis.

## Cell survival

Evidence indicates that IGF-I promotes cell survival by inhibiting apoptosis both *in vivo* and *in vitro*. These effects have been observed in neural progenitors and in multiple neuronal types such as cortical cells, motoneurons, Purkinje cells, or optic neural progenitor cells (Gago et al., 2003; Vicario-Abejón et al., 2004; Hodge et al., 2007; Croci et al., 2010; Aburto et al., 2012; Lunn et al., 2015). In the DG, the lack of IGF-I or IGF-IR causes a decrease in neuronal survival under basal conditions (Cheng et al., 2001; Lichtenwalner et al., 2006; Liu et al., 2009) or after ischemia (Liu et al., 2011) whereas IGF-I overexpression rescued neuronal survival in the lesioned HP (Carlson et al., 2014; Figure 1).

Moreover, IGF-I could prevent neuronal death in neurodegenerative diseases such as Alzheimer, regulating the accumulation of amyloid-β, and Tau proteins (Carro et al., 2002; Puche and Castilla-Cortázar, 2012). In fact, IGF-I enhances the transport of amyloid-β carrier proteins such as albumin and transthyretin, promoting its degradation (Carro et al., 2002). Moreover, this factor activates Akt which inhibits GSK3β, preventing Tau hyperphosporilation (Bondy and Cheng, 2004). In addition to the accumulation of amyloid-β and phosphorylated Tau proteins, the cognitive decline found in Alzheimer's patients might be attributable to decreased dentate gyrus neurogenesis. In contrast, an increase in IGF-I levels enhances neurogenesis (see below) and ameliorates the age-related cognitive malfunction in the brain. Therefore, restoring hippocampal neurogenesis by IGF-I may be a strategy for reversing age-related cerebral dysfunction. However, other studies have reported that IGF-I can promote the production of amyloid-β (Araki et al., 2009) and that knocking-out IGF-IR in neurons of a mouse model of Alzheimer's disease (AD) favors amyloid-β clearance probably by preserving autophagy and improves spatial memory (Gontier et al., 2015). This potential neuroprotective effect of reducing IGF-I/IGF-IR signaling has also been proposed for spinal muscular atrophy (SMA; Biondi et al., 2015). Therefore, the role of IGF-I in AD and motor neuron disease requires further investigation.

IGF-I can also prevent the gradual loss of other physiological functions associated with aging produced by oxidative stress and DNA damage, among others (Puche and Castilla-Cortázar, 2012). However, some *Igf-I* deficient mice, which have low levels of circulating IGF-I, exhibit an increased lifespan possibly due to alterations in energy metabolism and a transient enhancement in neurogenesis (Sun et al., 2005; Sun, 2006; Junnila et al., 2013; Chaker et al., 2015). These mice show an upregulation of local IGF-I levels in the hippocampus which could explain the increase in neurogenesis (Sun et al., 2005). All these data reveal that the effect of circulating and local IGF-I may be different but the full mechanisms have not been elucidated.

## Cell migration

IGF-I is also involved in the regulation of the migration of certain cell types. In neuroblastoma cell line cultures, IGF-I stimulates cell migration (Puglianiello et al., 2000; Russo et al., 2005). The first demonstration that IGF-I regulates cell migration and positioning *in vivo* was described by Hurtado-Chong et al. through *Igf-I* KO mice and explant cultures (Figure 1). These studies showed that IGF-I is necessary for the exit of neuroblasts from the SVZ to the OB and for the radial neuronal migration in the OB (Hurtado-Chong et al., 2009). These effects were mediated by the activation of the PI3K pathway and by phosphorylation of the reelin signal transducer, homolog 1 (Dab1; Hurtado-Chong et al., 2009). These findings indicate that IGF-I promotes adult neurogenesis not only by regulating NSC number and differentiation but also by directing neuronal positioning and migration (Figure 1). Successively, IGF-I has been related to the migration of doublecortin^+^ immature neurons in the SVZ-RMS, dorsal root ganglion neurons and cerebellar neurons in rodents, and neural crest cells in the zebrafish (Onuma et al., 2011; Xiang et al., 2011; Li et al., 2012; Maucksch et al., 2013).

## Neuronal generation, differentiation, and maturation

The IGF-I/IGF-IR system regulates the differentiation and maturation of neurons generated from NSCs and progenitors both during embryonic development and in the adult brain largely via the PI3K/Akt pathway (Aberg et al., 2000; Brooker et al., 2000; O'Kusky et al., 2000; Trejo et al., 2001; Vicario-Abejón et al., 2003; Otaegi et al., 2006; Carlson et al., 2014; Zhang et al., 2014; Yuan et al., 2015). Furthermore, IGF-I also influences the development of astrocytes and oligodendrocytes (Ye and D'Ercole, 2006; O'Kusky and Ye, 2012). Indeed, IGF-I promotes the differentiation of neural progenitors into mature oligodendrocytes that produce myelin (Carson et al., 1993; Ye et al., 1995; Gago et al., 2003; Hsieh et al., 2004) and stimulates the proliferation and differentiation of astrocytes under physiological conditions and after injury (Cao et al., 2003; Ye et al., 2004).

In *Igf-I* and *Igf-IR* KO mice, a reduction in the number of neurons during embryonic development and postnatal-adult neurogenesis in SVZ-OB and HP has been described (Baker et al., 1993; Liu et al., 1993, 2009; Powell-Braxton et al., 1993; Beck et al., 1995; Hurtado-Chong et al., 2009). When IGF-I was added to eOBSCs in culture, it produced an increase in the number of neurons, astrocytes and oligodendrocytes, whereas there was a decrease in the differentiation of eOBSCs isolated from the *Igf-I* KO mice (Vicario-Abejón et al., 2003; Otaegi et al., 2006). In the postnatal-adult OB of *Igf-I* KO animals, reductions in the number of interneuron populations were observed, possibly due to the altered neuroblast exit and migration from the SVZ, as mentioned above (Hurtado-Chong et al., 2009). By contrast, animals that overexpress *Igf-I* exhibit an increase in the number of neurons in the HP (O'Kusky et al., 2000; Popken et al., 2004; Carlson et al., 2014; Figure 1).

In addition to its role in the main neurogenic adult brain regions IGF-I also increases neurogenesis in the hypothalamus. After intra-cerebroventricular treatment with IGF-I, the number of neurons and astrocytes labeled with BrdU was significantly increased in the whole hypothalamus (Pérez-Martín et al., 2010). A similar effect of this growth factor was also found in hypothalamic cell cultures and explants (Torres-Aleman et al., 1990; Pérez-Martín et al., 2010).

IGF-I may regulate neuronal maturation, affecting axonal and dendritic growth, and establishing synapses in different brain areas independently of cell survival (O'Kusky et al., 2000; Cao et al., 2011; Figure 1). Thus, *Igf-I* KO animals have a lower development in the peripheral nerves (Gao et al., 1999), an altered innervation of the sensory cells of the organ of Corti (Camarero et al., 2001) and a lower density of spines in neurons of layers II-III of the cortex (Cheng et al., 2003). In the OB of *Igf-I* KO mice, the pattern of the axonal projections of sensory olfactory neurons is altered, because IGF-I acts as a chemoattractant for axonal growth cones (Scolnick et al., 2008). In the HP, IGF-I is involved in the establishment of neuronal polarity and the initial growth of the axonal cone, through the Akt pathway (Laurino et al., 2005; Sosa et al., 2006). Although the structure of the CB is preserved in E18.5 *Igf-I* KO mice (Vicario-Abejón et al., 2004), it has been shown that IGF-I promotes the establishment of cerebellar synapses whereas lack of IGF-I facilitates its removal during postnatal development (Kakizawa et al., 2003). Likewise, IGF-I overexpression in transgenic mouse promotes dendrite growth and synaptogenesis in the DG (O'Kusky et al., 2000; Carlson et al., 2014).

Exercise produces an increase in the IGF-I levels in adults which then stimulates an increase in the density of spines in the basal dendrites of CA1 pyramidal neurons but does not affect either the granule neurons in the GD or the CA3 pyramidal neurons (Glasper et al., 2010). Similarly, when IGF-I is administered by ventricular infusion no effect was observed in the number of synapses in CA3 (Poe et al., 2001). However, a decrease in the serum IGF-I levels causes a reduction of glutamatergic boutons in the HP (Trejo et al., 2007). This finding suggests that IGF-I entry to the HP can promote synapse formation and/or maintenance and as such can be beneficial for spatial learning and to reduce anxiety-like behavior (Llorens-Martín et al., 2010; Baldini et al., 2013). In contrast, it has been recently reported that the suppression of IGF-IR signaling in KO mice enhances olfactory function in aged males but not in females, adding new levels of complexity to the understanding of the role of IGF-I/IGF-IR in the regulation of neurogenesis, synaptogenesis and function in the adult brain (Chaker et al., 2015; Figure 1).

## Conclusions and future perspectives

The IGF-I affects the proliferation of progenitor cells, the survival of both progenitors and neurons, differentiation, and maturation of neurons in the neurogenic areas of the adult brain (Figure 1). In addition it regulates neuronal positioning and migration in the SVZ-OB. However, the studies performed have reported that the action of IGF-I signaling could be different or even opposite depending on the experimental approach used, the point in development and/or cell type affected. The production of cell-specific transgenic mouse lines, double KO for IGF-I and IGF-IR in combination with new technologies such as CRISPR-Cas9, optogenetics, and pharmacogenetics might contribute to the deeper understanding of the role and mechanisms of action of IGF-I/IGF-IR signaling during postnatal-adult neurogenesis. They also could help to elucidate the role of local and systemic IGF-I in this process and to identify new molecules regulated by this growth factor.

## Author contributions

VN Manuscript writing. ÇD Revision of the manuscript. CV Manuscript writing Financial support Final approval of the manuscript.

### Conflict of interest statement

The authors declare that the research was conducted in the absence of any commercial or financial relationships that could be construed as a potential conflict of interest.
